# Supplementary material for: The Association between TNF-α, IL-6, and Vitamin D Levels and COVID-19 Severity and Mortality: A Systematic Review and Meta-Analysis
Source: Pathogens. 2022 Feb 1;11(2):195. doi: 10.3390/pathogens11020195 (PMC8879207; doi:10.3390/pathogens11020195)
Supplement: Supplementary file 1 [file pathogens-11-00195-s001.zip › Supplementary Table S1. Study characteristics..pdf]

Supplementary Table S1. Study characteristics.

ID	Study, year	Country	Study design	Population	Severity criteria	n
				**TNF-α**		
**1**	Abers MS 2021 [32]	Italy	Cohort	COVID-19 patients	–	175
**2**	Hou H 2020 [33]	China	Cohort	COVID-19 patients	Guidelines of diagnosis and treatment for COVID-19 made by the Chinese National Health Commission	389
**3**	Liu G 2021 [34]	China	Cohort	COVID-19 patients	Not stated	296
**4**	Tian J 2020 [35]	China	Multicenter, retrospective cohort	COVID-19 patients with any type of malignant solid tumours and haematological malignancy	WHO guidelines, complemented by the Seventh Revised Trial Version of the COVID-19 Diagnosis and Treatment Guidance (2020) of China	232
**5**	Wang JH 2021 [36]	China	Single-center, retrospective cohort	COVID-19 patients	–	1135
**6**	Wang M 2021 [37]	China	Cohort	COVID-19 patients	Diagnosis and Treatment of Novel Coronavirus Patients (the Fifth Pilot Ed.)	211
**7**	Xie M 2021 [38]	China	Retrospective cohort	COVID-19 patients	2019 clinical practice guideline from American Thoracic Society (ATS) and Infectious Diseases Society of America (IDSA) for diagnosis and treatment of adults with community-acquired pneumonia and WHO criteria for severe COVID-19	280
**8**	Yang B 2021 [39]	China	Cohort	Patients with severe COVID-19	–	320
**9**	Zhu Z 2020 [40]	China	Single-center, retrospective cohort	COVID-19 patients	Guidelines for diagnosis and treatment for COVID-19 (Trail Version 6)	127
				**IL-6**		
**1**	Abers MS 2021 [32]	Italy	Cohort	COVID-19 patients	–	175
**10**	Avila-Nava A 2021 [41]	Mexico	Cohort	COVID-19 patients	–	38
**11**	Bai Y 2021 [42]	China	Retrospective cohort	COVID-19 patients	Guideline of diagnosis and management for COVID-19 (sixth edition) by the National Health Commission of China	3342
**12**	Bhadade R 2020 [43]	India	Cohort	Critically ill COVID-19 patients	–	373

**13**	Chen C 2020 [44]	China	Single-center, retrospective cohort	COVID-19 patients with comorbidities	–	132
**14**	Chen H 2021 [45]	China	Cohort	COVID-19 patients	–	855
**15**	Chen X 2020 [46]	China	Cohort	COVID-19 patients	New Coronavirus Pneumonia Diagnosis and Treatment Plan (trial version 6) by the National Health Committee of the People's Republic of China	102
**16**	Donoso-Navarro E 2021 [47]	Spain	Retrospective cohort	COVID-19 patients	–	546
**17**	Gao F 2021 [48]	China	Cohort	COVID-19 patients	Diagnosis and Treatment Protocol for Novel Coronavirus Pneumonia (Trial Version 7) by the National Health Commission & State Administration of Traditional Chinese Medicine	167
**18**	Gou L 2021 [49]	China	Retrospective cohort	Critically ill COVID-19 patients	–	114
**19**	Gu Y 2021 [14]	China	Single-center, retrospective cohort	COVID-19 patients admitted to the ICU	–	57
**2**	Hou H 2020 [33]	China	Cohort	COVID-19 patients	Guidelines of diagnosis and treatment for COVID-19 by the Chinese National Health Commission	389
**20**	Huang H 2021 [50]	China	Retrospective cohort	COVID-19 patients	Guidelines for the Diagnosis and Treatment for Novel Coronavirus Pneumonia (version 7) by the National Health Commission of the People's Republic of China	64
**21**	Laguna-Goya R 2020 [51]	Spain	Single-center, cohort	COVID-19 patients	–	501
**22**	Lavillegrand R 2021 [52]	France	Multicenter, cohort	COVID-19 patients admitted to the ICU	–	101
**23**	Li T 2020 [53]	China	Retrospective, cohort	COVID-19 patients not younger than 65 years old	American Thoracic Society guidelines for community-acquired pneumonia	312
**24**	Li T 2021 [54]	China	Retrospective cohort	COVID-19 patients	–	75
**3**	Liu G 2021 [34]	China	Cohort	COVID-19 patients	Not stated	296
**25**	Liu Q 2020 [55]	China	Cross-sectional	COVID-19 patients	Novel coronavirus pneumonia diagnosis and treatment plan (trial version 7)	84
**26**	Liu QQ 2020 [56]	China	Single-center, retrospective cohort	COVID-19 patients	–	308

**27**	Maeda T 2021 [57]	United States	Single-center, retrospective cohort	COVID-19 patients	–	224
**28**	Martinez-Ubristondo M 2020 [58]	Spain	Single-center, cross-sectional		–	165
**29**	Ruscica M 2021 [59]	Italy	Retrospective cohort	COVID-19 patients	–	97
**30**	Sai F 2021 [60]	China	Single-center, retrospective cohort	COVID-19 patients admitted to B-ICU	–	47
**4**	Tian J 2020 [35]	China	Multicenter, retrospective cohort	COVID-19 patients with any type of malignant solid tumours and haematological malignancy	WHO guidelines, complemented by the Seventh Revised Trial Version of the COVID-19 Diagnosis and Treatment Guidance (2020) of China	232
**31**	Trecarichi EM 2020 [61]	Italy	Single-center, retrospective cohort	COVID-19 patients	–	48
**5**	Wang JH 2021 [36]	China	Single-center, retrospective cohort	COVID-19 patients	–	1135
**6**	Wang M 2021 [37]	China	Cohort	COVID-19 patients	Diagnosis and Treatment of Novel Coronavirus Patients (the Fifth Pilot Ed.)	211
**32**	Wang Y 2020 [62]	China	Case-control	Children diagnosed with COVID-19	Diagnosis, treatment, and prevention of 2019 novel coronavirus infection in children: experts' consensus statement	43
**33**	Wu C 2020 [63]	China	Retrospective cohort	COVID-19 patients who developed ARDS	–	84
**7**	Xie M 2021 [38]	China	Retrospective cohort	COVID-19 patients	2019 clinical practice guideline from American Thoracic Society (ATS) and Infectious Diseases Society of America (IDSA) for diagnosis and treatment of adults with community-acquired pneumonia and WHO criteria	280
**8**	Yang B 2021 [39]	China	Cohort	Patients with severe COVID-19	–	320
**34**	Yi P 2020 [18]	China	Single-center, retrospective cohort	COVID-19 patients	Clinical management of 2019 nCoV guidelines by the National Health Commission of the People Republic of China	100

**35**	Zhao C 2021 [64]	China	Single-center, retrospective cohort	COVID-19 patients	Report of the WHO-China Joint Mission on COVID-19	172
**36**	Zhou F 2020 [65]	China	Multicenter, retrospective cohort	COVID-19 patients	–	191
**9**	Zhu Z 2020 [40]	China	Single-center, retrospective cohort	COVID-19 patients	Guidelines for diagnosis and treatment for COVID-19 (Trail Version 6)	127
**37**	Zhu Z 2021 [66]	China	Single-center, retrospective cohort	COVID-19 patients	National Diagnosis and Treatment Protocol for Novel Coronavirus Infection-Induced Pneumonia (6th Trial Version)	142
				**Vitamin D**		
**38**	AlSafar H 2021 [67]	United Arab Emirates	Multicenter, cohort	COVID-19 patients	–	464
**39**	Campi I 2021 [68]	Italy	Cohort	Patients with severe COVID-19	Severe criteria: Spontaneous oxygen saturation ≤93% and/or PaO2/FiO2 ratio < 300 mmHg) requiring invasive or non-invasive ventilation, with or without the presence of fever (>37.5°C) and other organ dysfunction	155
**40**	Cereda E 2021 [69]	Italy	Cohort	COVID-19 patients	–	129
**41**	De Smet D 2020 [70]	Belgium	Retrospective cohort	COVID-19 patients	–	186
**42**	Derakhshanian H 2021 [71]	Iran	Retrospective cohort	COVID-19 patients	–	290
**43**	Jahangirimehr A 2021 [72]	Iran	Cross-sectional	COVID-19 patients	Not stated	93
**44**	Jevalikar G 2021 [73]	India	Cross-sectional	COVID-19 patients	WHO ordinal scale for clinical improvement (OSCI)	410
**45**	Karahan S 2020 [74]	Turkey	Retrospective cohort	COVID-19 patients	Diagnosis and Treatment Protocol for Novel Coronavirus Pneumonia (Trial Version 7)	149
**46**	Lohia P 2021 [75]	United States	Retrospective cohort	COVID-19 patients	–	270
**47**	Pizzini A 2020 [76]	Austria	Cohort	COVID-19 patients	Severe criteria: patients requiring oxygen supply, respiratory support, or intensive care treatment	109
**48**	Vasheghani M 2021 [77]	Iran	Cross-sectional	COVID-19 patients	WHO criteria	508
