# Supplementary material for: The Association between TNF-α, IL-6, and Vitamin D Levels and COVID-19 Severity and Mortality: A Systematic Review and Meta-Analysis
Source: Pathogens. 2022 Feb 1;11(2):195. doi: 10.3390/pathogens11020195 (PMC8879207; doi:10.3390/pathogens11020195)
Supplement: Supplementary file 1 [file pathogens-11-00195-s001.zip › Supplementary Table S2. Studies investigating the association between TNF-a and severe COVID-19 with odds ratio values..pdf]

Supplementary Table ***S*****2.** Studies investigating the association between TNF-α and severe CoVID-19 with odds ratio values.

Study, year	Study design	Crude OR (95% CI)	p value	Adjusted OR (95% CI)	p value	Adjusted for
**Hou H 2020 [33]**	Cohort	–	–	1.0115 (0.9440–1.0837)	0.7450	IL-1β, IL-2R, IL-6, IL-8, IL-10, lymphocyte, neutrophil, PCT, IL-2R/lymphocytes, CRP, ferritin
**Liu G 2021 [34]**	Cohort	–	–	0.361 (0.138–0.941)	0.037	IFN-γ, IL-10, IL-2, IL-4, IL-6
**Tian J 2020 [35]**	Multicenter, retrospective cohort	1.22 (1.04–1.43)	0.015	1.22 (1.01–1.47)	0.037	Age, sex, comorbidities, tumour stage, cancer type, ECOG performance status, antitumour treatments, IL-2R, PCT, CRP, lymphocyte, lymphocytre count, CD3-CD19+ B-cell count, CD4+ T cells, CD3-CD16+CD56+ natural killer cells, leukocyte count, neutrophils, monocytes, LDH, albumin, albumin–globulin ratio, NT-proBNP, myoglobin, hs-cTnI, platelet count, activated partial thromboplastin time, prothrombin time, D-dimer
**Wang M 2021 [37]**	Cohort	–	–	0.783 (0.108–5.665)	0.808	Age, sex, duration from illness onset to admission, duration of hospitalization, temperature before admission, temperature at day 15 after admission, hypertension, diabetes, cardiovascular and cerebrovascular diseases, respiratory system diseases, white blood cell count, lymphocyte, alanine aminotransferase, aspartate aminotransferase, creatinine, IFN-g, IL-2, IL-4, IL-6, IL-10, CD3+ T cells, B cells, NK cells, CD4+ T cells, CD8+ T cells, CD4+/CD8+ ratio
**Xie M 2021 [38]**	Retrospective cohort	1.074 (1.013–1.139)	0.016	–	–	–
**Zhu Z 2020 [40]**	Single-center, retrospective cohort	0,917 (0.386–2.182)	0.845	–	–	–
