# Supplementary material for: The Association between TNF-α, IL-6, and Vitamin D Levels and COVID-19 Severity and Mortality: A Systematic Review and Meta-Analysis
Source: Pathogens. 2022 Feb 1;11(2):195. doi: 10.3390/pathogens11020195 (PMC8879207; doi:10.3390/pathogens11020195)
Supplement: Supplementary file 1 [file pathogens-11-00195-s001.zip › Supplementary Table S3. Studies investigating the association between TNF-a and COVID-19 mortality with hazard ratio values..pdf]

Supplementary Table S3. Studies investigating the association between TNF-α and CoVID-19 mortality with hazard ratio values.

Study, year	Study design	Crude HR (95% CI)	p value	Adjusted HR (95% CI)	p value	Adjusted for
**Abers MS 2021 [32]**	Cohort	1.26 (0.78–2.06)	0.3568	1.18 (0.81–1.71)	0.3819	Time to sampling, age, chronic kidney disease, and use of immunomodulatory medications prior to sample collection
**Wang JH 2021 [36]**	Single-center, retrospective cohort	1.085 (1.062–1.109)	<0.01	–	–	–
**Yang B 2021 [39]**	Cohort	1.044 (1.032–1.056)	<0.001	–	–	–
