# Supplementary material for: The Association between TNF-α, IL-6, and Vitamin D Levels and COVID-19 Severity and Mortality: A Systematic Review and Meta-Analysis
Source: Pathogens. 2022 Feb 1;11(2):195. doi: 10.3390/pathogens11020195 (PMC8879207; doi:10.3390/pathogens11020195)
Supplement: Supplementary file 1 [file pathogens-11-00195-s001.zip › Supplementary Table S4. Studies investigating the association between IL-6 and severe COVID-19 with odds ratio values..pdf]

Supplementary Table S4. Studies investigating the association between IL-6 and severe CoVID-19 with odds ratio values.

Study, year	Study design	Crude OR (95% CI)	p value	Adjusted OR (95% CI)	p value	Adjusted for
**Bai Y 2021 [42]**	Retrospective cohort	1.150 (1.001–1.321)	0.049	–	–	–
**Chen X 2020 [46]**	Cohort	–	–	1.099 (1.055–1.144)	0.000	CD4+ cells, CD8+ cells, T regs, B cells, NK cells, IL-10
**Gao F 2021 [48]**	Cohort	1.06 (1.03–1.09)	<0.001	1.04 (1.02–1.09)	0.002	Age, sex, overweight/obesity, type 2 diabetes, and hypertension
**Hou H 2020 [33]**	Cohort	–	–	1.0000 (0.9916–1,0085)	0.9936	IL-1b, IL-2R, IL-6, IL-8, IL-10, TNF-α, lymphocyte, neutrophil, PCT, IL-2R/lymphocytes, CRP, ferritin
**Huang H 2021 [50]**	Retrospective cohort	–	–	0.979 (0.917–1.045)	0.52	Age, ferritin, ALB, HGB, lymphocytes, glu, LDH, CRP, ESR
**Li T 2020 [53]**	Retrospective cohort	–	–	4.32 (2.07–7.13)	0.0016	Age, SOFA score, APACHEII score, platelet, d-dimer, creatinine, consolidation
**Liu G 2021 [34]**	Cohort	–	–	0.998 (0.996–1)	0.092	IFN-γ, IL-2, IL-4, IL-10, TNF-α
**Liu Q 2020 [55]**	Cross-sectional	–	–	1.281 (1.026–1.600)	0.029	Age, BMI, blood pressure, smoking history, coexisting disorder, white blood cell count, neutrophil, lymphocyte, NLR, PLR, CRP, SAA
**Tian J 2020 [35]**	Multicenter, retrospective cohort	1.03 (1.01–1.06)	0.014	1.03 (1.00–1.05)	0.019	Age, sex, comorbidities, tumour stage, cancer type, ECOG performance status, antitumour treatments, IL-2R, PCT, CRP, lymphocyte, lymphocytre count, CD3-CD19+ B-cell count, CD4+ T cells, CD3-CD16+CD56+ natural killer cells, leukocyte count, neutrophils, monocytes, LDH, albumin, albumin-globulin ratio, NT-proBNP, myoglobin, hs-cTnI, platelet count, activated partial thromboplastin time, prothrombin time, D-dimer
**Wang M 2021 [37]**	Cohort	–	–	1.227 (1.040–1.447)	0.015	Age, sex, duration from illness onset to admission, duration of hospitalization, temperature before admission, temperature at day 15 after admission, hypertension, diabetes, cardiovascular and cerebrovascular diseases, respiratory system diseases, white blood cell count, lymphocyte, alanine aminotransferase, aspartate aminotransferase, creatinine, IFN-g, IL-2, IL-4, IL-6, IL-10, CD3+ T cells, B cells, NK cells, CD4+ T cells, CD8+ T cells, CD4+/CD8+ ratio
**Wang Y 2020 [62]**	Case-control	1.05 (0.98–1.12)	0.14	–	–	–
**Xie M 2021 [38]**	Retrospective cohort	1.011 (1.005–1.018)	0.001	–	–	–

Study, year	Study design	Crude OR (95% CI)	p value	Adjusted OR (95% CI)	p value	Adjusted for
**Yi P 2020 [18]**	Single-center, retrospective cohort	–	–	1.005 (0.995–1.015)	0.307	Age, BMI, T cells, B cells, artificial liver support
**Zhao C 2021 [64]**	Single-center,retrospective cohort	1.115 (1.074–1.157)	<0.001	–	–	–
**Zhu Z 2020 [40]**	Single-center, retrospective cohort	1.086 (1.041–1.133)	<0.001	1.090 (1.040–1.147)	<0.001	Hypertension, CRP
**Zhu Z 2021 [66]**	Single-center, retrospective cohort	1.096 (1.050–1.144)	<0.001	1.097 (1.034–1.165)	0.002	Age, BMI, hypertension, neutrophil%, lymphocyte%, lymphocyte count, platelet count, fibrinogen, CRP, IL-6, IL-10, HDL-C, ApoA1, ALB, AST, and LDH
