# Supplementary material for: The Association between TNF-α, IL-6, and Vitamin D Levels and COVID-19 Severity and Mortality: A Systematic Review and Meta-Analysis
Source: Pathogens. 2022 Feb 1;11(2):195. doi: 10.3390/pathogens11020195 (PMC8879207; doi:10.3390/pathogens11020195)
Supplement: Supplementary file 1 [file pathogens-11-00195-s001.zip › Supplementary Table S5. Studies investigating the association between IL-6 and CoVID-19 mortality with odds ratio values..pdf]

Supplementary Table S5. Studies investigating the association between IL-6 and CoVID-19 mortality with odds ratio values.

Study, year	Study design	Crude OR (95% CI)	p value	Adjusted OR (95% CI)	p value	Adjusted for
**Bhadade R 2020 [43]**	Cohort	–	–	1.004 (1.001–1.008)	0.02441	Age, hypertension, DM, variable endotracheal intubation, PFR, SpO2, ferritin and D-dimer
**Chen C 2020 [44]**	Single-center, retrospective cohort	1.007 (0.971–1.044)	0.705	1.015 (0.948–1.086)	0.667	CHD, lymphocytes, monocytes, NLR, AST, PCT, SSA, D-dimer, FDP
**Gou L 2021 [49]**	Retrospective cohort	1.02 (1.01–1.04)	0,004	1.01 (1.00–1.03)	0.032	Age, gender, dyspnea, random blood glucose, eGFR, D-dimer, CRP, PT, sodium
**Gu Y 2021 [14]**	Single-center, retrospective cohort	1.009 (1.003–1.015)	0.003	1.013 (1.001–1.025)	0.028	PaO_2
**Laguna-Goya R 2020 [51]**	Single-center, cohort	1.008 (1.005–1.012)	<0.0001	–	–	–
**Lavillegrand R 2021 [52]**	Multicenter, cohort	–	–	2.20 (1.58–3.05)	<0.001	SOFA at ICU admission and time from symptom onset to dosage
**Liu QQ 2020 [56]**	Single-center, retrospective cohort	1.031 (1.022–1.040)	<0.001	1.013 (1,003–1,024)	0.015	Age, gender, comorbidities, disease severity, lymphocyte count, IL-2R, IL-8, IL-10, TNF-α
**Maeda T 2021 [57]**	Single-center, retrospective cohort	–	–	1.002 (1.000–1.003)	0.018	Age, D-dimer
**Martinez-Ubristondo M 2020 [58]**	Single-center, cross-sectional	–	–	9.81 (1.56–61.69)	0.02	Age, sex, Charlson Comorbidity Index Score, LDH, CRP, NLI, RDW, ferritin, exctraction day
**Sai F 2021 [60]**	Single-center, retrospective cohort	1.01 (1.00–1.02)	0.061	–	–	–
**Zhou F 2020 [65]**	Multicenter, retrospective cohort	1.12 (1.03–1.23)	0.0080	–	–	–
