# Supplementary material for: The Association between TNF-α, IL-6, and Vitamin D Levels and COVID-19 Severity and Mortality: A Systematic Review and Meta-Analysis
Source: Pathogens. 2022 Feb 1;11(2):195. doi: 10.3390/pathogens11020195 (PMC8879207; doi:10.3390/pathogens11020195)
Supplement: Supplementary file 1 [file pathogens-11-00195-s001.zip › Supplementary Table S6. Studies investigating the association between IL-6 and CoVID-19 mortality with hazard ratio values..pdf]

Supplementary Table S6. Studies investigating the association between IL-6 and CoVID-19 mortality with hazard ratio values.

Study, year	Study design	Crude HR (95% CI)	p value	Adjusted HR (95% CI)	p value	Adjusted for
**Abers MS 2021 [32]**	Cohort	1.87 (1.24–2.84)	0.0033	1.77 (1.24–2.52)	0.0015	Time to sampling, age, chronic kidney disease, and use of immunomodulatory medications prior to sample collection
**Avila-Nava A 2021 [41]**	Cohort	–	–	1.01 (1.003–1.020)	0.011	Age, gender, diabetes, hypertension
**Bai Y 2021 [42]**	Retrospective cohort	1.082 (1.055–1.110)	<0.001	1.085 (1.048–1.124)	<0.001	Age, sex, hypertension, diabetes, CHD, AF, CVD, COPD, CKD, malignancy, NLR, D-dimer, PTA, LDH, α-HBDH, ALB, DBIL, BUN, CysC, CRP
**Chen H 2021 [45]**	Cohort	–	–	1.001 (1.0003–1.002)	0.005	NEU, NLR, CRP, D-dimer, PT, MYO
**Donoso-Navarro E 2021 [47]**	Retrospective cohort	1.00094 (1.00054–1.0013)	<0.0001	1.0011 (1.001–1.0016)	<0.0001	Age, LDH, limfosit
**Li T 2021 [54]**	Retrospective cohort	1.02 (1.01–1.03)	<0.001	1.02 (1.01–1.04)	0.004	Age, CRP
**Ruscica M 2021 [59]**	Retrospective cohort	1.0033 (1.0014–1.0052)	0.001	–	–	–
**Trecarichi EM 2020 [61]**	Single-center, retrospective cohort	1.005 (1.002–1.008)	<0.001	1.005 (1.001–1.009)	0.007	Blood sodium level, lymphocytes, CVD other than hypertension
**Wang JH 2021 [36]**	Single-center, retrospective cohort	1.002 (1.002–1.002)	<0.001	–	–	–
**Wu C 2020 [36]**	Retrospective cohort	1.03 (1.01–1.05)	0.01	–	–	–
**Yang B 2021 [39]**	Cohort	1.001 (1.000–1.001)	<0.001	–	–	–
