# Supplementary material for: The Association between TNF-α, IL-6, and Vitamin D Levels and COVID-19 Severity and Mortality: A Systematic Review and Meta-Analysis
Source: Pathogens. 2022 Feb 1;11(2):195. doi: 10.3390/pathogens11020195 (PMC8879207; doi:10.3390/pathogens11020195)
Supplement: Supplementary file 1 [file pathogens-11-00195-s001.zip › Supplementary Table S7. Studies investigating the association between vitamin D levels and severe CoVID-19 with mean vitamin D values..pdf]

Supplementary Table S7. Studies investigating the association between vitamin D levels and severe CoVID-19 with mean vitamin D values.

Study, year	Study design	NOS score	Severe Mean±SD (ng/ml)	n	Non-severe Mean±SD (ng/ml)	n	p value
**Campi I 2021 [68]**	Cohort	6	18.2±11.4	103	30.3±8.4	52	<0,0001
**Jahangirimehr A 2021 [72]**	Cross-sectional	5	24.57±11.42	37	19.5379±10.753	56	0,077
**Jevalikar G 2021 [73]**	Cross-sectional	5	31.7±26.8	20	26.3±24.9	390	0,165
**Karahan S 2020 [74]**	Retrospective cohort	5	10.1±6.2	102	26.3±8.4	47	<0,001
**Pizzini A 2020 [76]**	Cohort	5	20±9.6	53	23.1714±9.8615	56	0,116
**Vasheghani M 2021 [77]**	Cross-sectional	5	25.8915±19.8631	223	30.734±23.1905	285	0,001
