# Supplementary material for: The Association between TNF-α, IL-6, and Vitamin D Levels and COVID-19 Severity and Mortality: A Systematic Review and Meta-Analysis
Source: Pathogens. 2022 Feb 1;11(2):195. doi: 10.3390/pathogens11020195 (PMC8879207; doi:10.3390/pathogens11020195)
Supplement: Supplementary file 1 [file pathogens-11-00195-s001.zip › Supplementary Table S8. Studies investigating the association between vitamin D deficiency and COVID-19 mortality..pdf]

Supplementary Table S8. Studies investigating the association between vitamin D deficiency[1] and COVID-19 mortality.

Study, year	Study design	Crude OR (95% CI)	p value	Adjusted OR (95% CI)	p value	Adjusted for
**AlSafar H 2021 [67]**	Multicenter cohort	0.94 (0.41–2.17)	0.89	1.71 (0.66–4.43)	0.27	Age, sex, and comorbidities
**Cereda E 2021 [69]**	Cohort	–	–	0.28 (0.09–0.99)	0.038	Age, sex, C-reactive protein, ischemic heart disease and severe pneumonia
**De Smet D 2020 [70]**	Retrospective cohort	–	–	3.87 (1.30–11.55)	0.0154	Age, sex, comorbidities, CT stage, CT severity score
**Derakhshanian H 2021 [71]**	Retrospective cohort	2.30 (1.25–4.26)	<0.05	2.0 (1.03–3.83)	<0.05	Age, sex
**Lohia P 2021 [75]**	Retrospective cohort	0.69 (0.39–1.24)	0.21	1.04 (0.55–1.97)	0.9	Age, sex, body mass index (BMI), and comorbidities

[1] Vitamin D deficiency is defined as 25(OH)D<20 ng/mL [78].
