# Supplementary material for: The Association between TNF-α, IL-6, and Vitamin D Levels and COVID-19 Severity and Mortality: A Systematic Review and Meta-Analysis
Source: Pathogens. 2022 Feb 1;11(2):195. doi: 10.3390/pathogens11020195 (PMC8879207; doi:10.3390/pathogens11020195)
Supplement: Supplementary file 1 [file pathogens-11-00195-s001.zip › Supplementary Text 1. Detailed search strategies and MeSH..pdf]

Supplementary File 1. Detailed search strategies and MeSH. Literature search was conducted on 6 August 2021 on PubMed, Cochrane, ProQuest and Google Scholar.

PubMed search strategy

(("TNF"[All Fields] OR "Tumor Necrosis Factor-alpha"[MeSH Terms]) OR ("interleukin 6"[MeSH Terms] OR "interleukin 6"[All Fields] OR "il 6"[All Fields]) OR ("vitamin d"[MeSH Terms] OR "vitamin d"[All Fields] OR "calcifediol"[MeSH Terms] OR "25(OH)D"[All Fields])) AND ("SARS-CoV-2"[MeSH Terms] OR "sars-cov-2"[All Fields] OR "covid"[All Fields] OR "covid-19"[MeSH Terms] OR "covid-19"[All Fields])

Cochrane search strategy

"Tumor Necrosis Factor-alpha" or Interleukin-6 or "Vitamin D" AND Covid-19

Used filter:

- Observational
- Case series/Case control/Cohort
- Diagnostic/Prognostic

ProQuest search strategy

("CoViD-19" OR "CoViD 19" OR "CoViD" OR "Coronavirus disease" OR "Coronavirus disease-2019" OR "Coronavirus disease 2019" OR "Coronavirus disease-19" OR "Coronavirus disease 19" OR "nCoV" OR "2019-nCoV" OR "2019 nCoV" OR "novel coronavirus" OR "2019-novel coronavirus" OR "2019 novel coronavirus" OR "SARS-CoV-2" OR "SARS CoV-2" OR "SARS CoV 2" OR "SARS-Coronavirus-2" OR "SARS Coronavirus-2" OR "SARS Coronavirus 2" OR "Severe Acute Respiratory Syndrome-Coronavirus-2" OR "Severe Acute Respiratory Syndrome Coronavirus-2" OR "Severe Acute Respiratory Syndrome Coronavirus 2") AND (("Tumor Necrosis Factor" OR "Tumor Necrosis Factor alpha" OR "Tumor Necrosis Factor-alpha" OR "Tumor Necrosis Factor α" OR "Tumor Necrosis Factor-α" OR "TNF" OR "TNF alpha" OR "TNF-alpha" OR "TNF α" OR "TNF-α") OR ("IL-6" OR "IL 6" OR "IL6" OR "Interleukin-6" OR "Interleukin 6" OR "Interleukin6") OR ("Vit D" OR "Vitamin D" OR "Cholecalciferol" OR "Ergocalciferol" OR "Calcifediol" OR "25-Hydroxyvitamin D 3" OR "25 Hydroxyvitamin D 3" OR "25-Hydroxycholecalciferol Monohydrate" OR "25 Hydroxycholecalciferol Monohydrate" OR "25-Hydroxyvitamin D3" OR "25 Hydroxyvitamin D3" OR "Calcidiol" OR "25-Hydroxycholecalciferol" OR "25 Hydroxycholecalciferol"))

Google Scholar search strategy

allintitle: Covid "TNF" OR "TNF-alpha" OR "IL-6" OR "Interleukin-6" OR "Vitamin D" OR "25(OH)D" OR "25-Hydroxyvitamin D"

MeSH for PubMed dan Cochrane

COVID-19

COVID 19

- COVID-19 Virus Disease
- COVID 19 Virus Disease
- COVID-19 Virus Diseases
- Disease, COVID-19 Virus
- Virus Disease, COVID-19
- COVID-19 Virus Infection
- COVID 19 Virus Infection
- COVID-19 Virus Infections
- Infection, COVID-19 Virus
- Virus Infection, COVID-19
- 2019-nCoV Infection
- 2019 nCoV Infection
- 2019-nCoV Infections
- Infection, 2019-nCoV
- Coronavirus Disease-19
- Coronavirus Disease 19
- 2019 Novel Coronavirus Disease
- 2019 Novel Coronavirus Infection
- 2019-nCoV Disease
- 2019 nCoV Disease
- 2019-nCoV Diseases
- Disease, 2019-nCoV
- COVID19
- Coronavirus Disease 2019
- Disease 2019, Coronavirus
- SARS Coronavirus 2 Infection
- SARS-CoV-2 Infection
- Infection, SARS-CoV-2
- SARS CoV 2 Infection
- SARS-CoV-2 Infections
- COVID-19 Pandemic
- COVID 19 Pandemic
- COVID-19 Pandemics
- Pandemic, COVID-19

SARS-CoV-2

- Coronavirus Disease 2019 Virus
- 2019 Novel Coronavirus
- 2019 Novel Coronaviruses
- Coronavirus, 2019 Novel
- Novel Coronavirus, 2019
- Wuhan Seafood Market Pneumonia Virus
- SARS-CoV-2 Virus
- SARS CoV 2 Virus
- SARS-CoV-2 Viruses
- Virus, SARS-CoV-2
- 2019-nCoV
- COVID-19 Virus
- COVID 19 Virus
- COVID-19 Viruses
- Virus, COVID-19
- Wuhan Coronavirus
- Coronavirus, Wuhan
- SARS Coronavirus 2
- Coronavirus 2, SARS
- Severe Acute Respiratory Syndrome Coronavirus

Tumor Necrosis Factor-alpha

- Tumor Necrosis Factor alpha
- Cachectin
- Cachectin-Tumor Necrosis Factor
- Cachectin Tumor Necrosis Factor
- Tumor Necrosis Factor Ligand Superfamily Member 2
- Tumor Necrosis Factor
- TNF Superfamily, Member 2
- TNFalpha
- TNF-alpha

Interleukin-6

- Interleukin 6
- IL6
- B-Cell Stimulatory Factor 2
- B-Cell Stimulatory Factor-2
- Differentiation Factor-2, B-Cell
- Differentiation Factor 2, B Cell
- B-Cell Differentiation Factor-2
- B Cell Differentiation Factor 2
- BSF-2
- Hybridoma Growth Factor
- Growth Factor, Hybridoma
- IFN-beta 2
- Plasmacytoma Growth Factor
- Growth Factor, Plasmacytoma
- Hepatocyte-Stimulating Factor
- Hepatocyte Stimulating Factor
- MGI-2
- Myeloid Differentiation-Inducing Protein
- Differentiation-Inducing Protein, Myeloid
- Myeloid Differentiation Inducing Protein
- B-Cell Differentiation Factor
- B Cell Differentiation Factor
- Differentiation Factor, B-Cell
- Differentiation Factor, B Cell
- IL-6
- Interferon beta-2
- Interferon beta 2
- beta-2, Interferon
- B Cell Stimulatory Factor-2
- B Cell Stimulatory Factor 2

Vitamin D

A vitamin that includes both CHOLECALCIFEROLS and ERGOCALCIFEROLS, which have the common effect of preventing or curing RICKETS in animals. It can also be viewed as a hormone since it can be formed in SKIN by action of ULTRAVIOLET RAYS upon the precursors, 7-dehydrocholesterol and ERGOSTEROL, and acts on VITAMIN D RECEPTORS to regulate CALCIUM in opposition to PARATHYROID HORMONE.
